# EAAT2 as a therapeutic research target in Alzheimer's disease: A systematic review

**DOI:** 10.3389/fnins.2022.952096

**Published:** 2022-08-10

**Authors:** Oliver W. G. Wood, Jason H. Y. Yeung, Richard L. M. Faull, Andrea Kwakowsky

**Affiliations:** ^1^Department of Anatomy and Medical Imaging, Faculty of Medical and Health Sciences, Centre for Brain Research, University of Auckland, Auckland, New Zealand; ^2^Pharmacology and Therapeutics, Galway Neuroscience Centre, School of Medicine, Ollscoil na Gaillimhe – University of Galway, Galway, Ireland

**Keywords:** glutamate transporter, EAAT2, hippocampus, Alzheimer's disease, human brain

## Abstract

Glutamate is the main excitatory neurotransmitter in the human central nervous system, responsible for a wide variety of normal physiological processes. Glutamatergic metabolism and its sequestration are tightly regulated in the normal human brain, and it has been demonstrated that dysregulation of the glutamatergic system can have wide-ranging effects both in acute brain injury and neurodegenerative diseases. The excitatory amino acid transporter 2 (EAAT2) is the dominant glutamatergic transporter in the human brain, responsible for efficient removal of glutamate from the synaptic cleft for recycling within glial cells. As such, it has a key role in maintaining excitatory-inhibitory homeostasis. Animal studies have demonstrated dysregulation or alterations of EAAT2 expression can have implications in neurodegenerative disorders. Despite extensive research into glutamatergic alterations in AD mouse models, there is a lack of studies examining the expression of EAAT2 within the AD human brain. In this systematic review, 29 articles were identified that either analyzed EAAT2 expression in the AD human brain or used a human-derived cell culture. Studies were inconclusive as to whether EAAT2 was upregulated or downregulated in AD. However, changes in localization and correlation between EAAT2 expression and symptomatology was noted. These findings implicate EAAT2 alterations as a key process in AD progression and highlight the need for further research into the characterization of EAAT2 processes in normal physiology and disease in human tissue and to identify compounds that can act as EAAT2 neuromodulators.

## Introduction

Alzheimer's disease (AD) is a progressive neurodegenerative condition that is the most common form of dementia, causing impairments in memory and cognitive function (Revi, 2020). Due to an aging population worldwide, the incidence of AD is predicted to increase rapidly in the coming years (Brookmeyer et al., 2007). As such, there is now an increasing need to develop a disease-modifying treatment or preventative approach for AD due to the social and economic burden associated with rising incidence. Many promising scientific interventions based on animal models have failed to translate into effective therapies in humans, raising doubts about the current leading theories behind AD's etiology (Tolar et al., 2019). Analyzing postmortem human brain tissue to determine cellular alterations to disease-relevant proteins is, therefore, a very valuable method to identify potential human-specific therapeutic targets for AD.

The main pathological hallmarks of AD are the aggregation of extracellular amyloid-beta (Aβ) plaques and intracellular tangles of the microtubule-associated protein tau (Bloom, 2014). These pathologies severely affect the hippocampal regions and the entorhinal cortex, causing neuronal cell loss and memory impairment (Braak and Braak, 1991). Neuronal death eventually spreads throughout the temporal cortex and to other brain regions in late disease (Braak and Braak, 1991). Other notable pathological features of AD include vascular deficits, mitochondrial damage, inflammation, and damage to neurotransmitter systems, which are contributing factors to extensive neuronal death (Šerý et al., 2013; Wang and Reddy, 2017; Hampel et al., 2018).

Glutamate is the primary excitatory neurotransmitter of the central nervous system (CNS), involved in a variety of vital functions, including neuronal communication and regulation of neuronal activity (Danbolt, 2001). Glutamate concentrations in the extracellular space are usually tightly regulated, and dysregulation can lead to overexcitation of postsynaptic neurons, potentially causing glutamate excitotoxicity. A growing body of evidence suggests glutamate dysfunction as a core component in the pathogenesis of neurodegenerative diseases, including AD (Hynd et al., 2004). Glutamate reuptake from the extracellular space is regulated by a class of transporters known as the excitatory amino acid transporters (EAATs).

There are five isoforms of the EAATs, with their expression being cell type and brain region specific (Malik and Willnow, 2019). The isoforms EAAT1 and EAAT2 are primarily expressed on astrocytes, with high expression in the cerebellum and throughout the entire brain, respectively (Kim et al., 2011; Pajarillo et al., 2019). EAAT3 is expressed throughout the brain, while EAAT4 expression is predominantly found in the cerebellum (Malik and Willnow, 2019). EAAT5 expression is mainly restricted to photoreceptors and bipolar cells in the retina (Arriza et al., 1997). EAAT2, encoded by the *SLC1A2* gene and also referred to by its rodent nomenclature, glutamate transporter 1 (GLT-1), is responsible for 90% of glutamate uptake in the mature brain (Kim et al., 2011; Pajarillo et al., 2019). Its expression is hence critical for controlling extracellular glutamate concentrations.

Several past animal studies have suggested that EAAT2 alteration occurs in AD. EAAT2 translocation has also been demonstrated in AD models and may play a role in EAAT2 dysfunction. EAAT2 haploinsufficiency has been shown to hasten cognitive decline in an AD mouse model, indicating that EAAT2 expression may play a critical factor in dictating AD progression (Mookherjee et al., 2011). Cross-linking of EAAT2 both *in vitro* and *ex vivo* in mouse hippocampal brain was found to alter astrocytic currents and neuronal EPSCs, although transporter function was preserved (Murphy-Royal et al., 2015). These findings support the notion that astrocytes are actively involved in shaping excitatory neurotransmission and highlight the importance of normal surface diffusion of glutamate transporters at the synapse in maintaining homeostasis and cellular signaling processes. In mouse hippocampal slices, Aβ_1−42_ induces rapid EAAT2 mislocalization and internalization in astrocytes, which leads to reduced cell surface expression and a marked reduction in glutamate reuptake from the extracellular space (Scimemi et al., 2013). Despite significant evidence pointing to glutamatergic dysfunction in AD, the majority of current literature has used mouse or other animal tissue studies to assess EAAT2 alterations and test therapeutic strategies for AD. However, only a limited amount of literature has used human tissue or cells to understand what may be happening to EAAT2 in AD.

Overall, this review aims to discuss the current evidence of EAAT2 expression and functional alteration in AD, a critical knowledge gap given that AD is ultimately a human disease. In addition, whether the existing literature warrants further research to investigate this glutamate transporter as a therapeutic target for the treatment or intervention of AD will be discussed.

## Methodology

**Figure 1:**
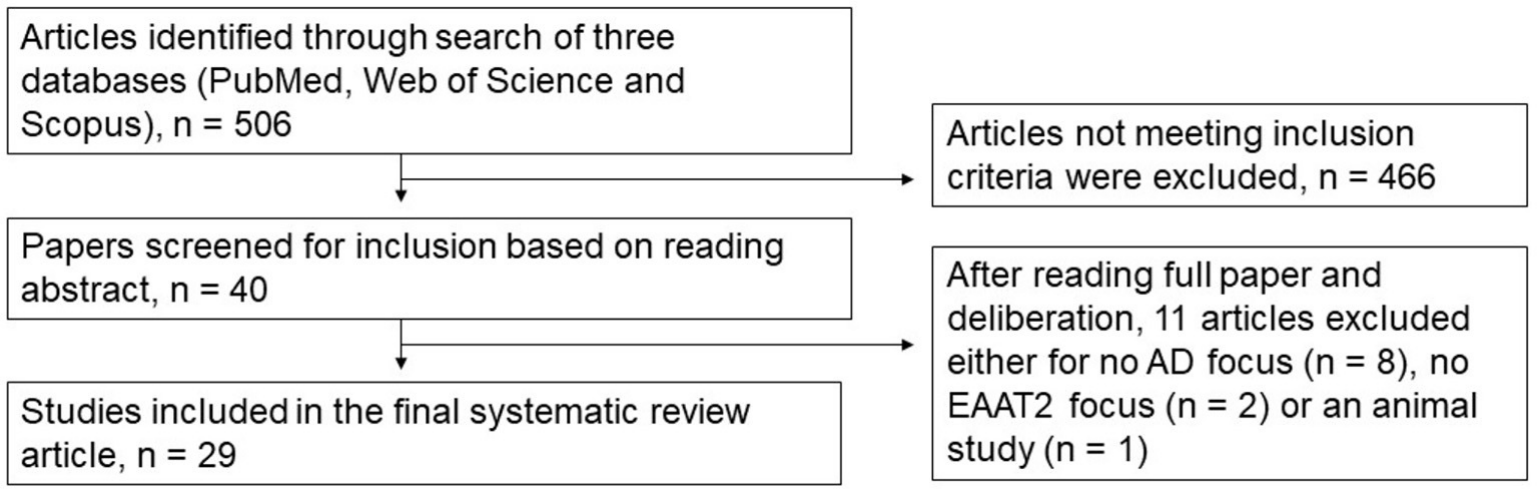
Flow diagram detailing the article selection process.

We performed a comprehensive literature search of PubMed, Web of Science and Scopus databases from 1991 through to 2022. Specific search terms used were Title, Abstract, Keywords/MeSH terms (“Excitatory Amino Acid Transporter 2” [Mesh] OR “SLC1A2 protein, human” [Supplementary Concept]) OR “Glt-1” OR “Glutamate transporter 1” OR “solute carrier family 1 member 2”) AND Title, Abstract, Keywords/MeSH terms: (Alzheimer Disease). Five hundred and six relevant articles were identified and collated in EndNote X8 (Clarivate Analytics, Philadelphia, PA, USA). Duplicates were removed using the Rayyan systematic review software (Rayyan QCRI, RRID:SCR_017584), and then sorted for inclusion or exclusion. Each abstract was screened independently by the first two authors to identify the articles that met our inclusion criteria of human-based studies with an EAAT2 focus. All authors discussed any conflicts to determine whether to include or exclude articles from this review. The inclusion criteria for this review were any EAAT2 study relevant to AD, including human-derived cultured cells, even those where EAAT2 may not have necessarily been the main focus of the study. Hence, animal-based studies or studies with no relevance to EAAT2 or AD were excluded. Twenty-nine articles were selected based on this criterion, and all authors agreed on this selection. The article selection process is shown in Figure 1, and the included studies are shown in Table 1 (postmortem studies) and Table 2 (cell cultures and other studies).

**Table 1:** Summary of human postmortem EAAT2 studies included in this systematic review.

**Study number**	**References**	**Aim/description**	**Main methodology/s**	**Subject/culture characteristics**	**Brain region examined**	**Main study findings (specific to EAAT2/AD)**	
1	Abdul et al. (2009)	Investigate whether nuclear factor of activated T-cells (NFAT) nuclear translocation is evident in AD -Examine EAAT2 expression in postmortem brain and determine whether activation of the NFAT pathway could be responsible for potential decreases in EAAT2	Western blot	12 control, 10 MCI, and 18 AD postmortem cases	Hippocampal sections—membrane fraction homogenate	EAAT2 protein expression significantly decreased in both MCI and AD cases relative to control	
2	Beckstrøm et al. (1999)	To determine whether reduced glutamate uptake may be relevant in AD by measuring levels of EAAT1 and EAAT2 in control and AD postmortem tissue	Western blot	10 control and 10 AD cases	Inferior temporal gyrus	Case variability in EAAT2 levels between both AD and control cases but no significant correlation seen between EAAT2 levels and AD diagnosis	
3	Flowers et al. (2001)	To examine whether EAAT2 splice variants retaining intron 7 or skipping exon 9 can be identified in the postmortem amyotrophic lateral sclerosis (ALS) motor cortex (with the inclusion of several AD cases)	TaqMan qPCR assay for quantification of EAAT2 mRNA transcripts in postmortem tissue	17 ALS cases, 7 AD cases, 19 control cases	Motor cortex, spinal cortex, anterior frontal gyrus, occipital cortex	The variant EAAT2 mRNA transcripts were detected in all cases across all brain regions. No significant difference in ratio of “variant” to “normal” transcripts in ALS or AD cases	
4	Garcia-Esparcia et al. (2018)	Examine GLT1/EAAT2 mRNA and protein expression in control, AD and dementia with Lewy body (DLB) cases	RT-Q-PCR and Western blot	39 middle aged control, 20 AD and 9 DLB cases	Frontal cortex, Brodmann area 8	Neither GLT-1/EAAT2 mRNA nor protein expression were significantly altered in AD cases compared to controls	
5	Hoshi et al. (2018)	To investigate the correlation between GLT-1 and aquaporin-4 (AQP4) expression in the human AD brain	Immunohistochemistry	5 control and 8 AD cases	Inferior, middle and superior temporal lobe	Significantly reduced GLT-1 expression in AD Two different patterns of GLT-1 and AQP4 in AD (i) uneven GLT-1 expression in neuropil but intense AQP4 expression (ii) co-expression of GLT-1 and AQP4 in plaque like structures	
6	Jacob et al. (2007)	Examine EAAT1/2 gene and protein expression in postmortem human brain	Q-RT-PCR and gene chip array	22 AD cases, 10 control cases	Gyrus frontalis medialis, hippocampus, and cerebellum	Gene chip array	SLC1A2 (EAAT2 gene) downregulated in AD cases (gyrus frontalis medialis), but no change in hippocampus or cerebellum
						mRNA (Q-RT-PCR)	EAAT2 mRNA expression decreased in hippocampus. No change in gyrus frontalis medialis or cerebellum
7	Kirvell et al. (2010)	To determine protein expression of glutamatergic components in vascular dementia postmortem tissue	Western blot	8 Control, 8 Vascular Dementia (VaD), 15 Mixed VaD/AD, 7 AD and 8 stroke no dementia (SND) cases.	Frontal cortex (Brodmann areas 9 and 20)	EAAT2 protein expression not significantly altered for any case group relative to control	
8	Kobayashi et al. (2018)	Examine potential neuroprotective role of astrocytes in AD—looking at GLT-1/EAAT2 expression	Immunohistochemistry	19 control cases (no dementia/AD pathology (N-N), 10 cases with AD pathology but no dementia (AD-N), and 18 with AD pathology and dementia (AD-D)	Entorhinal cortex (EC)	Significantly higher GLT-1 positive area in AD-N cases compared to those in the AD-D group for both layers I/II and III-VI of the EC Significantly lower GLT-1 positive area in AD-D cases compared to N-N cases in layer II/III of EC but not layers III–VI	
9	Lauderback et al. (2001)	Investigate the mechanisms behind decreased glutamate uptake in AD—exploring a connection between lipid peroxidation product 4-hydroxy-2-non-enal (HNE) and GLT-1	Immunoprecipitation and Western blot	7 Control and 4 AD cases	Inferior parietal lobule	Western blot analysis of HNE-modified proteins results in an increased GLT-1 immunoreactivity suggesting their co-expression in AD	
10	Li et al. (1997)	To determine which glutamate transporter subtype is most affected by AD and determine any correlations to abnormal amyloid precursor protein expression.	Western blot and RNase protection assay and Immunohistochemistry	4 Control and 12 AD cases	Frontal cortex	No significant change to EAAT2 mRNA levels in AD cases, but EAAT2 protein levels are significantly decreased.	
11	Poirel et al. (2018)	To investigate the expression of synaptic markers in dementia	Western blot	171 cases—(includes both control and AD cases). Cases grouped into clinical dementia ratings (CDR) 0 (38 cases), 0.5 (21), 1 (18), 2 (14), 3 (38), 4 (21), and 5 (21)	Frontal cortex (Brodmann area 9)	No expressional alterations to EAAT2 protein in AD cases and no correlation between the CDR and EAAT2 protein expression	
12	Pow and Cook (2009)	To analyze the expression of exon skipping variants of EAAT1-3 in AD postmortem tissue	Immunohistochemistry	3 control and 3 AD cases	Temporal cortex	Exon-9 skipping variant is evident in the AD temporal cortex. Glial cells and occasional neurons are labeled	
13	Rothstein et al. (1992)	To investigate whether glutamate transport is responsible for a suggested abnormal metabolism of glutamate in amyotrophic lateral sclerosis (ALS) and other neurodegenerative diseases	Quantification of sodium dependent glutamate uptake from homogenized postmortem tissue using radioactive hydrogen isotope	17 control cases (no neurological disease), 13 amyotrophic lateral sclerosis (ALS) cases, 12 Huntington's disease cases and 15 AD cases	Spinal cord, motor cortex, somatosensory cortex, striatum, and hippocampus	Mean V_max_ (indicative of relative density of EAAT2) and K_t_ (affinity) values for high affinity glutamate transport were not changed in AD tissue compared to control for any region, suggesting no change to EAAT2 expression in AD. (somatosensory cortex not examined for AD cases).	
14	Sasaki et al. (2009)	To examine the relationship between phosphorylated tau and EAAT2 in AD and other tauopathies	Western blot Immunoprecipitation Immunohistochemistry	3 Control, 4 AD, 2 progressive supranuclear palsy (PSP) and 2 corticobasal degeneration (CBD) cases	Temporal cortex	EAAT2 co-precipitated with p-tau in disease cases but not control EAAT2 partially co-localizes with tau in AD tissue Detergent insoluble EAAT2 evident in AD cases	
15	Scott et al. (2011)	To examine EAAT2 expression in the AD postmortem brain, including alternatively spliced variants	RT-PCR	15 Control, 12 AD and 10 AD/Lewy body disease cases	Inferior frontal, Inferior temporal, primary motor, and occipital cortices	Decreased mRNA expression of wild-type EAAT2 in all analyzed areas in AD. Increased wild-type EAAT2 mRNA copy number with increasing pathological severity in AD (irrespective of brain region) Splice variant EAAT2b mRNA levels slightly decreased in AD tissue Both exon 7 and 9 skipping variants of EAAT2 show an increase in mRNA copy number with increasing pathological severity in AD	
16	Simpson et al. (2010)	To investigate astrocyte pathology and relationship to AD related changes	Immunohistochemistry	42 AD cases (8 definite, 14 probable, 20 possible based on CERAD criteria)	Lateral temporal cortex	Variability in staining pattern of EAAT2 and GFAP visible in AD—grouped into three categories, minimal, moderate or extensive immunoreactivity Significant inverse relationship between EAAT2 and GFAP staining	
17	Thai (2002)	Investigate whether EAAT2 may be involved in AD related neuronal changes	Immunohistochemistry	21 brains from autopsy cases—split into three groups—cognitively normal, AD-related pathology but cognitively normal and AD	Various brain regions (cerebellum/brainstem, medial temporal lobe, and frontal cortices/basal ganglia)	EAAT2 immunoreactive neurons found in AD, but not control cases	
18	Thal et al. (2010)	To investigate whether neuropathological features and perivascular protein expression differ between cases with cerebral amyloid angiopathy (CAA) and AD	Immunohistochemistry	71 AD cases 309 Control cases. CAA cases determined from analysis of vascular deposition of amyloid-beta	Occipital cortex −20 cortical vessels in layers II and III studied for association with EAAT2 positive astrocytic processes	Expression of perivascular EAAT2—decreased in astrocytes of AD cases with capillary CAA but not in AD cases lacking capillary CAA and control cases	
19	Tian et al. (2010)	To investigate whether EAAT2's association with lipid rafts is disrupted in AD cases *via* Western blotting	Lipid raft preparation Western blot	6 AD and 11 AD cases	Frontal cortex (Brodmann areas 9 and 10)	EAAT2 protein levels significantly decreased in AD frontal cortex Significantly decreased association of EAAT2 with lipid rafts in AD cases over control	
20	Woltjer et al. (2010)	To investigate whether accumulation of detergent-soluble EAAT2 is related to the CDR in AD cases	ELISA and Immunohistochemistry	Hippocampus −22 AD, 14 CDR 0.5 cases, 13 control, and 4 PD Frontal cortex 55 AD, 23 CDR 0.5 cases, 20 control and 4 PD	Hippocampus and frontal cortex	Detergent insoluble EAAT2 significantly increased in the hippocampus and frontal cortex of AD patients relative to control	
21	Yeung et al. (2021)	Investigation of EAAT2 protein expression in the AD postmortem medial temporal lobe	Quantitative fluorescent Immunohistochemistry	7 control and 6 AD cases	Hippocampus, subiculum, entorhinal cortex, and superior temporal gyrus	No significant quantitative changes in EAAT2 expression in AD in any region investigated Higher EAAT2 staining was evident in the neuropil of AD cases	

**Table 2:** Summary of cell culture, gene expression and other human EAAT2 studies included in this systematic review.

**Study number**	**References**	**Aim/description**	**Main methodology**	**Subject/culture characteristics**	**Brain region examined**	**Main study findings (specific to EAAT2/AD)**
22	Batarseh et al. (2017)	To examine whether oleocanthal (in extra virgin olive oil) can rescue GLT-1 deficits in protein expression related to oligomeric Aβ exposure in astrocytes	Western blot	CCF-STTG1 human astrocytoma cell line	Cultured cells	Aβo exposure significantly reduced GLT-1 expression, which was rescued with oleocanthal co-treatment
23	Han et al. (2016)	Examine whether EAAT2 mRNA and protein expression is disturbed by Aβ exposure in human astrocytes and whether insulin could offer protection	RT-PCR and Western Blot	Human astrocyte cell line (HA-1800)	Cultured cells	EAAT2 mRNA levels not altered after Aβ exposure, but protein expression is significantly decreased. Insulin co-treatment rescues EAAT2 deficit
24	Liang et al. (2002)	To explore whether estrogen treatment can restore deficient glutamate transporter activity in cultured human astrocytes (while also investigating GLT-1 and GLAST expression)	Western blot	Primary human astrocytes—prepared from 8 control and 8 AD donors	Cultured cells	AD astrocytes express less GLT-1 protein than control astrocytes
25	Meng and Mei (2019)	To determine whether certain genes are differentially expressed in AD	Bioinformatics study	RNA-seq expression data from human brain samples from four separate studies from the AMP-AD programme. 1. ROSMAP study (640 samples) 2. MSBB study (938 samples) 3. MayoPilot study (93 samples) 4. MayoBB study (556 samples) Cases grouped as control and AD samples based on clinical annotation from the four different projects. After quality control evaluations, 1,045 AD and 622 control RNA-seq samples were used to calculate the Spearman correlation between AD and normal gene pairs	ROSMAP—Sequence data is from dorsolateral prefrontal cortex, posterior cingulate cortex and head of caudate nucleus. MSBB study—four different brain regions with separate sequence data, parahippocampal gyrus, inferior frontal gyrus, superior temporal gyrus and frontal pole Mayo studies—sequence data is from temporal cortex and cerebellum	SLC1A2 (EAAT2 gene) is not differentially expressed in AD (not significantly different between AD and control)
26	Meng et al. (2020)	Explore the correlation between EAAT2 and ADORA2A serum levels in AD—use as a biomarker?	EAAT2 ELISA detection kit	60 healthy controls, 68 AD cases	Serum expression of EAAT2 and ADORA2A analyzed *via* ELISA	The AD group showed significantly lower EAAT2 serum levels compared to control. Severity of AD showed a significant negative correlation to serum EAAT2 level
27	Sharma et al. (2019)	To investigate the differential roles that astrocytic and neuronal EAAT2 deficiencies might play in AD primarily using mice models. Also compare gene expression profiles of mice to the human condition	Data mining of GSE48350 from the Gene Expression Omnibus repository. Generated lists of differentially expressed genes in human AD and aging. Extent of overlap between astrocytic and neuronal deficient EAAT2 mice and humans determined using rank-rank hypergeometric overlap (RRHO)	N/A	N/A	Astrocytic EAAT2 deficient, but not neuronal EAAT2 deficient mice show overlap with the gene expression profiles in human AD and aging
28	Zoia et al. (2004)	Examine whether platelet expression of glutamate transporters is altered in AD	Western blotting and RT-PCR	60 control and 10 AD patients (blood samples taken)	Platelets prepared from patient blood	No change in EAAT2 protein or mRNA expression in platelets in AD
29	Zoia et al. (2011)	To explore glutamate uptake and transporter expression in human fibroblasts after exposure to non-fibrillar Aβ_1−42_	Semi quantitative RT-PCR and Western blotting	Human fibroblasts prepared from skin biopsies of 15 healthy controls and 6 AD patients	Cultured cells	No changes to protein or mRNA EAAT2 expression after exposure to non-fibrillar Aβ_1−42_

## Summary of findings

Only a small group of studies in current literature have investigated the expression of EAAT2 in human postmortem tissue, and this is reflected by the relatively small number of papers that met the inclusion criteria for this review. These articles have yielded contradictory findings regarding EAAT2 expression in AD, even for some studies investigating the same brain regions. In addition, a handful of studies have utilized human-derived cultured cells to investigate EAAT2 dysfunction in AD. Overall, despite a small sample size, it would appear these results broadly support findings from animal work and suggest a role for Aβ induced dysfunction of EAAT2 in AD.

### Postmortem human tissue

#### Temporal cortex and hippocampus

Some research has concluded EAAT2 protein expression is reduced in AD postmortem hippocampal (Abdul et al., 2009) and temporal gyrus tissue (Hoshi et al., 2018). However, a recently published study by our laboratory group revealed no quantitative EAAT2 alterations in the hippocampus, subiculum, entorhinal cortex, or superior temporal gyrus (Yeung et al., 2021). Despite this, changes to the expression pattern of EAAT2 were evident, with higher immunoreactivity noticeable in the neuropil (Yeung et al., 2021). The notion that EAAT2 expression level may not be altered in AD is further supported by earlier evidence from Beckstrøm et al. (1999), who found varying EAAT2 levels in the inferior temporal gyrus for both control and AD cases, but no clear association with AD. However, Jacob et al. (2007) and Scott et al. (2011) provided evidence for a decrease in EAAT2 mRNA expression in the AD temporal cortex, complicating current literature. Further results include those from Simpson et al. (2010), who reported variability in EAAT2 staining patterns between AD cases and a significant inverse relationship between GFAP and EAAT2 expression. Rothstein et al. (1992) reported no change to high-affinity glutamate transport. In addition, Woltjer et al. (2010) found an increase in detergent-insoluble EAAT2 in both the AD hippocampus and frontal cortex.

Interestingly, one study reported that cognitively intact patients with AD-related pathology in the entorhinal cortex showed greater GLT-1 immunoreactivity and greater mRNA expression than those with symptoms of dementia (Kobayashi et al., 2018). This is a fascinating result as no other study in postmortem tissue has revealed that different levels of EAAT2 expression might be correlated to clinical symptoms in the presence of AD pathology.

#### Frontal cortex

Similar to the temporal cortex, there is conflicting information in the frontal cortex regarding potential changes to the expression of EAAT2 in AD. Several studies have suggested no changes to EAAT2 expression in AD and dementia (Kirvell et al., 2010; Garcia-Esparcia et al., 2018; Poirel et al., 2018). Others have reported a decreased mRNA (Jacob et al., 2007; Tian et al., 2010; Scott et al., 2011) and protein (Li et al., 1997) EAAT2 expression. Tian et al. (2010) also found evidence for a decreased association of EAAT2 with lipid rafts in AD cases, potentially indicating a reduction in membrane expression.

#### Other anatomical areas

Compared to the temporal and frontal cortices, the expression of EAAT2 in other brain areas has not been as extensively studied. Jacob et al. (2007) found no evidence of EAAT2 alteration in AD postmortem cerebellar tissue. In the parietal cortex, there is evidence that a lipid peroxidation product 4-hydroxy-2-nonenal (HNE) is co-expressed with EAAT2, potentially indicating oxidative damage and loss of transporter function (Lauderback et al., 2001). However, the significance of this finding and whether it is true for other brain regions needs further investigation.

#### Alternatively-spliced variants and neuronal expression of EAAT2 in Alzheimer's disease

Several studies have researched whether alternatively spliced variants of the EAAT2 protein have altered expression and subsequently play a role in the pathophysiology of AD. Scott et al. (2011) found that both the exon seven and exon nine skipping variants have an increased mRNA copy number with increasing pathological severity in AD cases. Neuronal expression of the exon nine skipping EAAT2 variant has been reported in AD postmortem tissue *via* immunohistochemistry (Pow and Cook, 2009). However, Flowers et al. (2001) found no change in the expression of intron seven retaining and exon nine skipping transcripts in AD, although this was a small sample of AD cases. Thai (2002) also provided evidence for EAAT2 accumulation in neurons in AD but not in control postmortem brains, but it is uncertain if this is the full length or a splice variant of EAAT2.

### Human cell culture studies

Several studies utilizing human-derived cultured cells have also been used to investigate potential alterations to EAAT2 expression in AD. Batarseh et al. (2017) found reduced GLT-1 protein expression in a human astrocyte cell line after Aβ exposure. Another study confirmed this result but revealed that while EAAT2 protein levels are decreased after Aβ exposure, there was no change to EAAT2 mRNA transcript levels (Han et al., 2016). This discrepancy between mRNA and protein EAAT2 levels has also been documented in AD postmortem tissue (Li et al., 1997; Kobayashi et al., 2018). Thus, it is postulated that modifications to EAAT2 protein expression in AD result from posttranslational disturbances.

Zoia et al. (2011) found no change to both EAAT2 mRNA and protein in fibroblasts derived from AD patients after Aβ exposure, but this is perhaps unrelated to what is happening to astrocytic expression of EAAT2 in AD. Liang et al. (2002) also found a decrease in GLT-1 protein expression in astrocytes cultured from AD patients. Therefore, these studies suggest that exposure to Aβ may induce a loss of the EAAT2 protein in astrocytes, while mRNA levels are not affected. However, the functional consequences for glutamate uptake are still unknown and the exact reason behind the mRNA-protein discrepancy needs more clarification.

### Other relevant studies

One study found that serum levels of EAAT2 were lower in AD patients than in control, and this decrease was inversely correlated to the clinical symptoms of AD (Meng et al., 2020). This finding indicates that ELISA analysis of EAAT2 levels in patient serum may be a potential diagnostic tool for AD, although more work is needed to establish whether this change is specific to AD alone.

Only one paper from the reviewed studies used a bioinformatic approach to investigate EAAT2 gene expression in AD. Meng and Mei (2019) found that by analyzing RNA-seq datasets from four different projects, *SLC1A2* was not a differentially expressed gene in AD. Multiple brain regions were represented in the mRNAseq data including the dorsolateral prefrontal cortex, parahippocampal gyrus, inferior frontal gyrus, temporal cortex, and cerebellum (see Table 2, study 25 for full details).

## Discussion

There are several possible explanations for the differing results regarding the expression of both EAAT2 mRNA and protein in AD postmortem tissue from the studies included in this review. These results could simply indicate that there is high patient variability of EAAT2 mRNA or protein expression in AD. Additionally, low sample sizes often seen in human studies, mainly due to tissue availability, can produce potentially variable and confounding results. For example, some studies included in this review only used three AD and three control cases (Pow and Cook, 2009). This sample size does not give confidence that these results are replicable across the population. Other factors to consider when looking at the results of postmortem studies are the heterogeneity of AD, stage of disease, age and sex differences, postmortem delay, and medication effects. At this stage, it is unclear how these factors may influence the EAAT2 mRNA and protein expression as well as preservation in the postmortem human brain, an important fact to consider when interpreting variable results between studies. A final point is that different EAAT2/GLT-1 antibodies between studies, likely targeting different regions of the protein, may also be playing a role in conflicting results.

### EAAT2 protein expression

Based on early previous evidence from radiolabeling, an ~30% reduction in [^3^H] aspartate binding was revealed in the postmortem AD brain, signaling a significant dysfunction in glutamate uptake (Masliah et al., 1996). This is likely to play a role in AD progression, with implications such as glutamate excitotoxicity, a well-documented concept in the literature (Conway, 2020; Manisha et al., 2020). However, how this might relate to EAAT2 expression in the human AD brain is still unclear. Animal studies have suggested that Aβ may be responsible for decreases in EAAT2 expression (Takahashi et al., 2015; Huang et al., 2018). In addition, amyloid-beta administration has been shown to result in the mislocalization of EAAT2 (Manisha et al., 2020). However, it is uncertain if this is the reason behind EAAT2 alterations (both expressional and qualitative) previously documented in AD postmortem tissue (Li et al., 1997; Abdul et al., 2009; Hoshi et al., 2018; Yeung et al., 2021). After Aβ treatment, HNE and GLT-1 conjugation and impairments in glutamate uptake were reported in rat cortical synaptosomes (Keller et al., 1997). This result potentially indicates that the same changes seen with HNE and GLT-1 co-precipitation in the human brain by Lauderback et al. (2001) are related to Aβ exposure. More work is needed to establish the exact cellular mechanisms behind Aβ induced dysfunction of EAAT2. Interestingly, certain studies within the same brain region have shown contradictory results regarding EAAT2 protein expression (Abdul et al., 2009; Yeung et al., 2021). A possible explanation could be the methodology used. Abdul et al. (2009) reported a significant decrease in EAAT2 protein expression in the AD hippocampus *via* analysis of the membrane fraction in a Western blot. This result possibly indicates that EAAT2 expression on astrocytic membranes is reduced in AD, which seems to be in agreement with Tian et al.'s (2010) finding of a significantly decreased association between lipid rafts and EAAT2 in AD. However, our previous study reported no significant quantitative differences in EAAT2 staining in hippocampal regions. Despite this, we did note higher EAAT2 staining in the neuropil that appeared to show less co-localization with GFAP stained astrocytic main branches, also perhaps indicative of EAAT2 astrocytic loss (Yeung et al., 2021). Interestingly, this EAAT2 expression pattern in AD cases (higher staining in the neuropil) appears similar to immunohistochemical images from the hippocampus published by Li et al. (1997). However, no quantitative data was provided in this study for hippocampal areas, raising validity concerns about this comparison. Further analysis is needed to determine if this expression pattern seen in the AD hippocampus represents a loss of EAAT2 on astrocytic membranes or whether this is simply EAAT2 staining on fine astrocytic processes that were not stained with GFAP. Animal work also supports that the underlying pathophysiology is related more to a displacement of transporters than reduced transporter expression, as total EAAT2 expression from the same whole-cell protein lysates was not changed by Aβ_1−42_ in mouse hippocampal slices (Scimemi et al., 2013). More research is needed to validate this theory of EAAT2 astrocytic displacement, investigating if EAAT2 cell surface trafficking pathways are altered in AD, as this can have functional implications on glutamate uptake and thus play a key role in neurodegeneration.

### EAAT2 mRNA expression

Both cell culture models and human postmortem studies have reported a loss of EAAT2 protein expression in AD, despite no disturbances to EAAT2 mRNA expression (Li et al., 1997; Han et al., 2016). It suggests that any modification to EAAT2 protein expression by Aβ may be posttranslational, but how this may occur is uncertain. However, two studies included in this review reported decreased EAAT2 mRNA in AD tissue (Jacob et al., 2007; Scott et al., 2011), highlighting the contradictory nature of these results, and the inability to rely on the current literature available to accurately determine the processes impacting on EAAT2 mRNA and protein expression in AD. It is also possible that both protein and mRNA expression are differentially affected by AD in distinct brain regions. However, the theory that EAAT2 mRNA expression may be preserved in AD, is supported by findings that *SLC1A2* gene expression is not altered in AD (Meng and Mei, 2019). Although the RNAseq data used was from multiple brain regions, it cannot be ruled out that there might be subtle anatomical and layer-specific variations to *SLC1A2* expression in response to AD. Further work is needed to confirm EAAT2 mRNA and protein expression in AD, given the contradictory results reported in the above discussed studies.

### EAAT2 solubility and aggregation

A decrease in EAAT2 astrocytic protein expression was noted in an AD model by Scimemi et al. (2013), who found Aβ_1−42_ can cause rapid mislocalization of EAAT2 cell surface expression on astrocytes. Additionally, Woltjer et al. (2010) revealed that detergent-insoluble EAAT2 in the hippocampus and frontal cortex of AD patients was higher than control cases, indicating possible aggregation of EAAT2 in AD. Further analysis, though, revealed no evidence of an association between EAAT2 and amyloid plaques (Woltjer et al., 2010). Perhaps EAAT2 expression is lost from astrocytic membranes in AD and forms insoluble structures, independent of Aβ plaques which could explain these results. The increased EAAT2 in the neuropil seen in our earlier study (Yeung et al., 2021) could also be indicative of aggregation. Sasaki et al. (2009) noted that high molecular weight EAAT2 was detected in the detergent-insoluble fraction of their analyses, again potentially suggesting aggregation of EAAT2 in AD. However, it is uncertain if this is insoluble EAAT2 alone or its co-association with tau or amyloid aggregates. This study did indicate EAAT2 co-precipitates with p-tau (Sasaki et al., 2009), so EAAT2 and tau co-aggregates may occur in AD. Additionally, Hoshi et al. (2018) reported plaque-like staining of EAAT2 in AD, which was co-expressed with aquaporin-4. These results seem to suggest EAAT2 protein expression may be lost from astrocytic membranes in AD and form aggregates. However, it is not clear where EAAT2 expression might be translocated to, or what consequences this may have for progression of disease or neurodegeneration.

### EAAT2 as a neuroprotective factor

There is evidence that the preservation of EAAT2 expression on astrocytes may be neuroprotective in the presence of AD pathology (Kobayashi et al., 2018). Lower immunoreactivity of GLT-1 was seen in entorhinal layers I/II and III–VI of cases with dementia (AD-D) compared to control, but those with AD pathology but no dementia (AD-N) showed no change to GLT-1 positive area over control (Kobayashi et al., 2018). This result implies that the maintenance of EAAT2 expression in the presence of AD pathology is neuroprotective and may even be a key factor in determining AD symptomatology, more so than other established pathological AD changes. This study is the only postmortem human evidence supporting the maintenance of or increased EAAT2 expression as a therapeutic target in AD. A study investigating potential differences in serum expression of EAAT2 between control and AD patients found significantly lower expression of EAAT2 in their AD group (Meng et al., 2020). Interestingly, when AD subjects were grouped into “mild,” “moderate,” and “severe” based on the severity of the disease, severe subjects had significantly lower serum expression of EAAT2, and a significant negative correlation between severity and EAAT2 serum expression was noted (Meng et al., 2020). Although it cannot be certain that serum levels of EAAT2 reflect the expression of the protein in the brain, these results broadly seem to support the Kobayashi study. Numerous research from animal studies further supports that higher EAAT2 transporter activity in the presence of AD pathology may be neuroprotective and these results are discussed in the subsequent therapeutics chapter.

### EAAT2 splice variants

Alternatively-spliced variants of the EAAT2 protein (intron seven retaining and exon nine skipping) have been documented in both control and AD postmortem tissue, although there was no alteration to their mRNA expression noted in neurological disease (Flowers et al., 2001). However, conflicting evidence suggests an increased mRNA expression of exon 7 and 9 skipping splice variants in AD cases, which was found to correlate with increased pathological severity (Scott et al., 2011). Gebhardt et al. (2010) found evidence for the formation of heteromeric wild-type EAAT2 and variant protein complexes in HEK293 cells. In addition, it was found that cells co-transfected with wild-type and variant EAAT2 showed a reduction in glutamate-dependent activity measured by EC_50_ and Hill coefficients (Gebhardt et al., 2010). It remains to be determined if this reduction is relevant in the human brain. Due to the small number of studies in current literature, more investigation is needed into the splice variants of EAAT2 and their relationship to AD.

### Neuronal expression of EAAT2

Neuronal expression of EAAT2 is still a highly debated area of research. Historically, EAAT2 expression and thus glutamate uptake was thought to be localized exclusively to astrocytes (Milton et al., 1997; Rimmele and Rosenberg, 2016). More recent work though has indicated a small amount of EAAT2 immunoreactivity is likely to be localized to neurons (Roberts et al., 2014). In one immunohistochemistry study, in both the white and gray matter, ~80% of identifiable immunoreactivity for EAAT2 was deemed attributable to astrocytic processes. The remaining immunoreactivity was seen in axon terminals, dendrites or was unidentifiable (Melone et al., 2011). However, the exact differences in physiological function between neuronal and astrocytic EAAT2 are still not well-understood, although EAAT2 expressed on astrocytic processes perform the vast majority of glutamate reuptake and is also responsible for protection against glutamate excitotoxicity (Petr et al., 2015; Danbolt et al., 2016). The study from Sharma et al. (2019) found that an astrocytic deficiency of EAAT2 in mice, rather than neuronal, is more correlated with gene expression profiles seen in aging and AD, indicating that it is likely astrocytic EAAT2 that is affected in AD. Without further study though, it cannot be conclusively ruled out that changes to neuronal EAAT2 may have a role to play in AD.

It has been suggested that neuronal accumulation of EAAT2 may be relevant to neuron degeneration in AD (Pow and Cook, 2009). Some studies have found EAAT2 immunoreactive neurons throughout many brain regions in AD (Thai, 2002). Others have suggested that splice variants of EAAT2 may become translated in stressed neurons and have provided evidence for their accumulation in AD (Pow and Cook, 2009). However, it is inconclusive whether these results reflect true neuronal staining, as neither of these studies included a neuronal marker. Therefore, these immunoreactive “neurons” described in AD tissue may be astrocytic labeling wrapping around neurons or staining of other neuronal-like structures. Future research needs to establish whether EAAT2 accumulation in neurons is relevant in AD and whether this could contribute to AD pathophysiology.

### EAAT2 transporter activity in Alzheimer's disease

There are several previous studies in the literature that have investigated the functionality of the EAAT2 transporter in postmortem brains by assessing glutamate uptake. Some of these studies have shown a reduction in glutamate uptake in postmortem AD tissue (Hardy et al., 1987; Procter et al., 1988), while others have found no change (Beckstrøm et al., 1999). An article from Petr et al. (2015), suggests that these discrepancies may be due to the methodology used. From using both astrocytic and neuronal specific knockouts of GLT-1 in mice, they noted that interestingly, neuronal but not astrocytic GLT-1/EAAT2 made a significant contribution to glutamate uptake measured in synaptosomes (Petr et al., 2015). This is paradoxical, as neuronal or axon terminal EAAT2 only accounts for ~20% of its total labeling (Melone et al., 2011), so the astrocytic contribution to uptake would be expected to be much greater. The authors postulate that tissue homogenization may differentially affect astrocytic and neuronal EAAT2 function, with the former being suggested to be localized to leaky compartments and thus is unable to play a role in glutamate uptake. An alternative explanation is that the astrocytic EAAT2 (or a large proportion) may not be functional, perhaps due to environmental context (Petr et al., 2015). Reconstituting synaptosomes in liposomes removed this bias in their study, a step which they suggest may have caused discrepancies in earlier studies, as without reconstitution, astrocytic EAAT2 activity may be completely removed in a fashion unrelated to AD (i.e., unable to contribute to glutamate uptake or not functional; Petr et al., 2015). It is also entirely plausible that the differing results reported in literature may just reflect low sample sizes and variability associated with human tissue use. Moreover, the Petr et al. (2015) study is ultimately an animal study, so we cannot be sure this effect also occurs in human tissue. Nevertheless, these findings highlight the need to carefully consider methodology when comparing results between studies measuring glutamate uptake in AD, as well as the complicated nature of assessing glutamate uptake from both astrocytes and neurons in postmortem tissue.

### Evidence for EAAT2 modulation as a valid therapeutic target for the treatment of AD

Modulation of EAATs may have positive therapeutic effects, with their ability to alter glutamate levels providing a logical link in managing glutamate excitotoxicity in the AD brain. Upregulation of EAAT2 has been shown to reduce excitotoxic damage seen in a variety of acute and chronic neurological diseases, and over-expression of EAAT3 appears to protect against toxicity by decreasing the levels of extracellular glutamate (Lewerenz et al., 2006; Sheldon and Robinson, 2007). Furthermore, beneficial effects of memantine, an NMDAR antagonist approved by the FDA for the symptomatic management of AD, indicate that a reduction in circulating glutamate may provide another avenue toward reducing overexcitation of glutamatergic neurons (Plosker and Lyseng-Williamson, 2005).

There has been a focus on identifying and testing novel compounds with EAAT2-enhancing properties to identify their effect on AD models. Synthetic compound LDN/OSU-0212320 improved cognitive functions in the APP_Sw,Ind_ mouse model by increasing EAAT2 expression through translational activation (Takahashi et al., 2015). Other methods of EAAT2 modulation have also been explored. A recent medical hypothesis published by Manisha et al. (2020) described increasing EAAT2 activity by promoting glutamate ligand binding through allosteric modulator GT949. Enhancement of glutamate ligand binding is effective in promoting EAAT2 activity independent of substrate pharmacokinetics (Kortagere et al., 2018). Improving EAAT2 activity through increased allosteric binding might be an effective mechanism through which AD-associated EAAT2 dysfunction can be ameliorated.

The antibiotic ceftriaxone is a known activator of EAAT2 transcription, and several studies have investigated whether treatment with this compound may be beneficial in animal models of AD (Zumkehr et al., 2015; Fan et al., 2018; Hamidi et al., 2019). These results suggest that the upregulation of EAAT2 has beneficial effects on cognition and neuronal activity in animal models of AD and is a promising therapeutic target. Human research shows that increased EAAT2 expression is associated with cognitively intact subjects with AD pathology (Kobayashi et al., 2018), potentially validating enhancement of EAAT2 activity as a good therapeutic approach for AD. However, as an antibiotic compound, there are likely to be numerous off-target effects and problems with delivering this drug across the blood-brain barrier. Human research from Lee et al. (2008) has indicated that the NF-kB pathway is responsible for the ceftriaxone dependent upregulation of EAAT2. Perhaps this information could be used to design or search for treatment options that can act similarly to ceftriaxone to induce EAAT2 upregulation. However, more clarification and research is needed before upregulating EAAT2 can be considered a valid strategy to treat the human condition.

Additional novel and well-established compounds have also been examined for their potential affinity and synergistic properties in modulating the EAAT2 transporter. For example, the vitamin E derivative trolox is effective in ameliorating mislocalization of astrocytic EAAT2 in mice exposed to Aβ (Scimemi et al., 2013). Medications used for non-neurodegenerative conditions, such as minocycline, dexamethasone, and histamine, have been shown to have neuroprotective effects through the upregulation of EAAT2 (Fontana, 2015). Examination of current therapeutics used for other neurodegenerative disorders might also be useful as potential drugs for AD. Riluzole, currently approved for the management of amyotrophic lateral sclerosis, has been shown to promote activation of EAAT2 translation and reducing excitotoxic cellular injury in cultured neurons (Kong et al., 2014). Overall, EAAT2 modulation does appear to have neuroprotective effects in AD models, although further studies will be required to explore the applicability of these compounds and their suitability as disease-modifying therapies for AD. A short summary of EAAT2 modulation strategies in the current literature are outlined in Table 3.

**Table 3:** A summary of possible avenues to restore EAAT2 function and treat Alzheimer's disease.

**EAAT2 modulation strategies to increase glutamate clearance and treat AD**	**References**
Translational activation of EAAT2	Kong et al., 2014; Takahashi et al., 2015; Zumkehr et al., 2015; Fan et al., 2018; Hamidi et al., 2019; Manisha et al., 2020
Allosteric modulation to promote glutamate binding	Manisha et al., 2020
Restore aberrant mislocalization of EAAT2	Scimemi et al., 2013

## Future implications and conclusion

Overall, in this review, we highlight the contradictory nature of existing literature and indicate a potential for maintained EAAT2 astrocytic expression as a potential therapeutic option in AD. First and foremost, though, more research is needed to truly establish what happens to EAAT2 expression on astrocytic membranes in AD, how this may be related to Aβ and tau pathologies, and what effect this may have on excitotoxic glutamate disturbances. In addition, the potential contribution of these changes to AD's clinical symptoms and progression has to be explored. EAAT2 modulation may still be a valid therapeutic strategy for AD treatment or prevention, but these unknowns need answering before compounds aiming to restore EAAT2 function or expression are trialed extensively in clinics to treat AD.

## Data availability statement

The original contributions presented in the study are included in the article/Supplementary material, further inquiries can be directed to the corresponding author/s.

## Author contributions

OW, JY, and AK: conceptualization, methodology, and writing—original draft preparation. OW, JY, AK, and RF: writing—review and editing. AK and RF: supervision and funding acquisition. AK: project administration. All authors have read and agreed to the published version of the manuscript.

## Funding

This work was supported by Alzheimer's New Zealand Charitable Trust (AK; 370836), Alzheimer's New Zealand (AK; 3718869), Freemasons New Zealand (AK; 3719321), Aotearoa Foundation, Centre for Brain Research and University of Auckland (AK; 3705579), and Health Research Council of New Zealand (RLF; 3627373).

## Conflict of interest

The authors declare that the research was conducted in the absence of any commercial or financial relationships that could be construed as a potential conflict of interest.

## Publisher's note

All claims expressed in this article are solely those of the authors and do not necessarily represent those of their affiliated organizations, or those of the publisher, the editors and the reviewers. Any product that may be evaluated in this article, or claim that may be made by its manufacturer, is not guaranteed or endorsed by the publisher.
